# γ-Glutamyltransferase and Breast Cancer Risk Beyond Alcohol Consumption and Other Life Style Factors – A Pooled Cohort Analysis

**DOI:** 10.1371/journal.pone.0149122

**Published:** 2016-02-10

**Authors:** Oliver Preyer, Dorthe Johansen, Jessica Holly, Tanja Stocks, Alfonso Pompella, Gabriele Nagel, Hans Concin, Hanno Ulmer, Nicole Concin

**Affiliations:** 1 Agency for Preventive and Social Medicine, Bregenz, Vorarlberg, Austria; 2 Surgery Research Unit, Lund University, Malmoe, Sweden; 3 Department of Obstetrics and Gynaecology, Innsbruck Medical University, Innsbruck, Austria; 4 Lund University Diabetes Centre, Diabetes and Cardiovascular Disease—Genetic Epidemiology Department of Clinical Sciences Malmö, CRC, Malmoe, Sweden; 5 Department of Experimental Pathology, University of Pisa, Medical School, Pisa, Italy; 6 Institute of Epidemiology and Medical Biometry, Ulm University, Ulm, Germany; 7 Department of Medical Statistics, Informatics and Health Economics, Innsbruck Medical University, Innsbruck, Austria; Institute of Psychiatry, UNITED KINGDOM

## Abstract

**Objective:**

Elevated γ-Glutamyltransferase serum levels are associated with increased risk of overall cancer incidence and several site-specific malignancies. In the present prospective study we report on the associations of serum γ-Glutamyltransferase with the risk of breast cancer in a pooled population-based cohort considering established life style risk factors.

**Methods:**

Two cohorts were included in the present study, i.e. the Vorarlberg (n = 97,268) and the Malmoe cohort (n = 9,790). Cox proportional hazards regression models were fitted to estimate HRs for risk of breast cancer.

**Results:**

In multivariate analysis adjusted for age, body mass index and smoking status, women with γ-Glutamyltransferase levels in the top quartile were at significantly higher risk for breast cancer compared to women in the lowest quartile (HR 1.21, 95% CI 1.09 to 1.35; p = 0.005). In the subgroup analysis of the Malmoe cohort, γ-Glutamyltransferase remained an independent risk factor for breast cancer when additionally considering alcohol intake. A statistically significant increase in risk was seen in women with γ-Glutamyltransferase-levels in the top versus lowest quartile in a multivariate model adjusted for age, body mass index, smoking status, physical activity, parity, oral contraceptive-use and alcohol consumption (HR 1.37, 95% CI 1.11–1.69, p = 0.006).

**Conclusion:**

Our findings identified γ-Glutamyltransferase as an independent risk factor for breast cancer beyond the consumption of alcohol and other life style risk factors.

## Introduction

γ-Glutamyltransferase (GGT) is a key enzyme in glutathione (glutamyl-cysteinyl-glycine; GSH) metabolism. It catalyses the degradation of extracellular GSH, thus providing component amino acids that are then available for further intracellular GSH production. GSH functions as the major antioxidant of the cell, neutralizing reactive oxygen compounds and free radicals [1]. GSH catabolism is modulated uniquely by the enzyme GGT. It hydrolyses the γ-Glutamyl bond between glutamate and cysteine. Oxidative stress conditions can induce the expression of GGT as shown in experimental studies [2–4]. On the other hand, it has been repeatedly shown that GGT activity itself can give rise to prooxidant reactions [5].

For decades the determination of serum GGT levels has represented a reliable marker for evaluation of liver dysfunction, particularly in connection with alcohol consumption [1]. However, recent epidemiologic studies have shown elevated GGT to independently influence morbidity and mortality from causes other than liver disease [6–9]. GGT plays a role in major diseases such as diabetes, metabolic syndrome, and cardiovascular disease [7, 10]. Also, elevated GGT levels are implicated in increased risk of overall cancer incidence and several site-specific malignancies in women and men [11, 12]. Strasak et al. showed an increased risk of cancer in digestive organs, respiratory system/intrathoracic organs, urinary organs (in men), and lymphoid and hematopoietic cancers (in women). Furthermore, in this study GGT levels significantly impacted the risk for pooled female malignancies (breast and female genital malignancies), although the specific female tumour sites were not discriminated [11]. With respect to breast cancer, two previous studies have focused on the association of GGT levels and breast cancer risk. Fentiman et al. identified high GGT levels to positively correlate with breast cancer incidence in premenopausal patients only, while Hemelrijck et al. established GGT as a breast cancer risk factor in general [13, 14]. Interpretation of these studies is hampered by lack or limited knowledge on established breast cancer risk factors, particularly with respect to life style factors. Besides, a recent study has investigated the prognostic impact of pre-therapeutic GGT levels in primary metastatic breast cancer patients and established GGT serum levels as a novel prognostic factor [15].

In this prospective study we report on the associations of serum GGT levels with the risk of breast cancer in a pooled population-based cohort of a total of 107,058 women considered established risk factors for breast cancer including age, body mass index (BMI), smoking status, physical activity, parity, oral contraceptive (OC)-use and alcohol consumption. Two cohorts were included into the present study, i.e. the Vorarlberg cohort, Austria’s westernmost federal state, and the Malmoe cohort, Sweden’s third largest city. Cross-sectional correlates of GGT with various breast cancer risk factors were performed in the Malmoe subgroup.

## Materials and Methods

### Study populations

The Vorarlberg Health Monitoring and Promotion Program (VHM&PP) is one of the world´s largest population-based risk factor surveillance programs. The cohort was initiated in 1985 and is conducted by the Agency for Social and Preventive Medicine in Vorarlberg, the westernmost federal state of Austria. All adults in the region were invited to participate through a combination of different measures including written invitations, television, radio, and newspaper reports. Participants were enrolled continuously from 1985 through 2004. Follow-up was determined based on subject’s home addresses using a recall system of written biennial re-invitation letters. Loss to follow-up (e.g., due to migration) was <1%.

Sociodemographic data were recorded, and trained local physicians and internists conducted a voluntary physician examination regularly in a standardized manner. Costs were covered by the participant´s (compulsory) health insurance. A more detailed description of the program methodology has been reported elsewhere [16].

Between 1985 and 2004, 98,995 female Vorarlberg residents (ages>18 yrs.) were enrolled in the VHM&PP. We excluded 1,676 participants (1.7%) with missing or incomplete data on GGT at enrolment or with history of malignancies prior to enrolment. To eliminate possible effects of preclinical cancer by producing/altering GGT, we further excluded participants with baseline GGT serum values >600 units/L (n = 51), resulting in a total of 97,268 women eligible for analyses for the current investigation.

The second cohort included into the present study is the Malmoe Preventive Project (MPP). This project was set up in 1974 as an integrated institute within the Department of Medicine at Malmoe University Hospital, Sweden. The main objective of the MPP was to screen a middle-aged population for risk factors such as cardiovascular diseases, diabetes mellitus and alcoholism, and thereby develop methods–on an individual patient basis–for early detection, health education and prevention of a number of diseases and risk factors [17]. Captured anamnestic data included e.g. age, BMI, smoking status, physical activity, parity, OC-use, alcohol consumption and treatment for nervous or mental disorders. Between 1974 and 1994, complete birth-year cohorts of registered residents in Malmoe were invited by letter to a health screening investigation. Women born in 1926, 1928, 1930–1938, 1941 and in 1949 were invited. All 10,902 female residents were enrolled in the MPP. Exclusions were made for participants with missing or incomplete data on GGT at enrolment, or with a history of malignancies prior to enrolment (n = 1098). To eliminate possible effects of preclinical cancer by producing/altering GGT, we further excluded participants with baseline GGT serum values >600 units/L (n = 14), resulting in a total of 9,790 women eligible for analyses for the current investigation.

The participants of both cohorts signed informed consents to have personal data stored and processed. For this study, institutional board approval was obtained by the Ethics Committees in the respective countries (The Ethics Committee of Vorarlberg, decision 2006-6/2; The Ethics Committee at Lund University, decision LU-828-02).

### Measurement of GGT

Two central laboratories undergoing regular internal and external quality procedures enzymatically determined serum GGT concentrations on blood samples in the Vorarlberg cohort. It has been shown that GGT displays a considerable intra-individual stability and strong “tracking” pattern. Within 60 to 204 min after venous blood sample collection from a cubital vein, serum was obtained by centrifugation for 15 minutes at 4000 rotations per minute. Subsequently, GGT concentrations were measured until 2002 at 25°C, after that at 37°C and were given as units per Litre (U/L). A standard laboratory method, using Ƴ-glutamyl-Ƥ-nitroanilin as a substrate, was used by Malmoe University Hospital to analyse plasma-GGT on blood samples in the Malmoe cohort [18].

For analysis all GGT measurements were recalculated to 37°C and given as U/L. According to previously published data levels of GGT 18 U/L were regarded as normal high or elevated [19].

### Statistical analysis

Cox proportional hazards regression analysis was used to estimate hazard ratios (HRs) and their 95% confidence intervals (CIs) for the association of GGT with breast cancer incidence. Follow-up for a woman started at the date of her health examination and ended at invasive breast cancer diagnosis or at censoring. Censoring events were death, cancer diagnosis at other site, end of study, loss to follow-up and emigration.

First, we computed univariate HRs with 95% CIs using baseline GGT levels divided into quartiles (groups ≤13 U/L, 14–17 U/L, 18–25 U/L and ≥26 U/L) in the pooled Vorarlberg and Malmoe cohort. Due to rounded measurements of GGT frequencies in the quartiles do not match exactly 25%. Second, we fitted Cox models using GGT adjusting for age, BMI and smoking status (model I). For the Malmoe cohort only, a model was computed with alcohol consumption as an additional covariate, further adjusting for age, BMI, smoking status, physical activity, parity and OC-use (model II and model III). We evaluated whether the GGT-breast cancer relationship was modified by age, BMI, smoking status and alcohol status by testing interaction effects in the respective Cox models. The proportional hazards assumption was checked using Schoenfeld residuals and visual inspection of the hazard plots. The visual inspection revealed proportionality of hazard functions. Results of significance testing on Schoenfeld residuals showed that the proportional hazard assumption was fulfilled for each covariate included in the Cox models (all p-values>0.05).

In the Malmoe cohort, cross-sectional covariates of GGT with established and potential risk factors for breast cancer were evaluated using a multiple linear regression model. GGT as a dependent variable was log-transformed in this analysis. Binary predictor variables were included as 0/1 indicator variables (0 = no, 1 = yes) into the multiple linear regression analysis.

Alcohol consumption was estimated on the basis of a questionnaire in the Malmoe cohort. Several questions addressed self-reported alcohol consumption. The question “Do you mainly drink alcohol at weekends or public holidays” showed a high degree of completeness and validity due to the indirect way of questioning and was therefore selected for analysis. All statistical analyses were conducted using IBM SPSS 20.0 statistical software.

## Results

### Characteristics of study population

Demographic and clinical characteristics of the study population are shown in Table 1. The Vorarlberg cohort comprised of 97,268 women, the Malmoe cohort of 9,790 women, respectively. Median follow-up time was 16.7 years with a total of 1,497,730 person-years at risk and 24.3 years with a total of 219,789 person-years at risk in the Vorarlberg and the Malmoe cohort, respectively. In the Vorarlberg cohort 2,436 (2.5%) women and in the Malmoe cohort 761 women (7.8%) developed breast cancer in the course of follow-up.

**Table 1:** Characteristics of the Vorarlberg and the Malmoe cohort and of the pooled study population.

Study population	Vorarlberg		Malmoe		Total	
	N (%)	Mean (± SD); Median	N (%)	Mean (± SD); Median	N (%)	Mean (± SD); Median
**Participants**	97,268		9,790		107,058	
**Follow-up (years)**	97,268	15.4 (± 6.4); 16.7	9,790	22.5 (±7.8); 24.3	107,058	16.1 (±6.8); 17.5
**Total person-years at risk**	1,497,730		219,789		1,717,519	
**GGT (units/L, based on 37°C)**	97,268	24 (±33); 18	9,790	24 (±30); 17	107,058	24 (±33); 17
**Age (completed years)**	97,268	41 (±16); 38	9,790	49 (±7); 52	107,058	42 (±15); 40
**Body mass index (kg/m**^**2**^**)**	97,242	24.3 (±4.7); 23.3	9,787	24.4 (±4.3); 23.6	107,029	24.3 (±4.6); 23.3
Smoking status*						
Never	75,654 (77.8)		4,095 (44.4)		79,749 (74.9)	
Former	3,192 (3.3)		1,941 (21)		5,133 (4.8)	
Current	18,422 (18.9)		3,191 (34.6)		21,613 (20.3)	
Physical activity*						
**No**	n.a.		2198 (22.5)		2198 (22.5)	
**Yes**	n.a.		7555 (77.5)		7555 (77.5)	
Parity*						
0	n.a.		1152 (11.8)		1152 (11.8)	
1	n.a.		6023 (61.8)		6023 (61.8)	
OC-use*						
No	n.a.		8841 (90.7)		8841 (90.7)	
Yes	n.a.		908 (9.3)		908 (9.3)	
Alcohol consumption**						
No	n.a.		7,301 (75.8)		7,301 (75.8)	
Yes	n.a.		2,325 (24.2)		2,325 (24.2)	
Work status*						
Unemployed / retired	30,465 (33.6)		4,255 (59.3)		34,720 (35.5)	
Employed	60,189 (66.4)		2,917 (40.7)		63,106 (64.5)	
Work type*						
Manual	33,458 (40.2)		4.067 (46.9)		37,525 (40.8)	
Non-manual	49,800 (59.8)		4,603 (53.1)		54,403 (59.2)	
Civil status*						
Single	16,299 (17.4)		889 (9.1)		17,188 (16.6)	
Married	59,787 (63.8)		6,699 (68.6)		66,486 (64.2)	
Divorced	7,450 (7.9)		1,689 (17.3)		9,139 (8.8)	
Widowed	10,205 (10.9)		483 (4.9)		10,688 (10.3)	
**Status end of FU**						
Breast cancer	2,436 (2.5)		761 (7.8)		3,197 (3)	
Censored other cancer	6,042 (6.2)		1,820 (18.6)		7,862 (7.3)	
Censored death	7,273 (7.5)		914 (9.3)		8,187 (7.6)	
Censored loss to FU	0 (0)		135 (1.4)		135 (1.4)	
Censored end of study	81,517 (83.8)		6,160 (62.9)		87,677 (81.9)	

SD standard deviation, y years, kg kilograms, m^2^ square meters, °C degrees Celsius, FU follow-up, L Litres, n.a. not available

* Numbers do not add to total N because of missing values

** Alcohol consumption based on question”Do you mainly drink alcohol at weekends or public holidays?”

Median GGT level was 18 U/L and 17 U/L in the Vorarlberg and the Malmoe cohort, respectively. Mean age at study entry was 41 (±16) years and 49 (± 7) years in the Vorarlberg and Malmoe cohort, respectively. Median body mass index (BMI) was similar in both cohorts (23.3 kg/m^2^ in the Vorarlberg and 23.6 kg/m^2^ in the Malmoe cohort). Smoking status was available in both cohorts, distinguishing between never, former, and current smokers in 77.8%, 3.3% and 18.9% in the Vorarlberg cohort and in 44.4%, 21% and 34.6% in the Malmoe cohort, respectively.

### Crude and adjusted risk estimates of breast cancer incidence

The crude (univariate model) and adjusted risk (multivariate model I) estimates of breast cancer incidence in the pooled Vorarlberg and Malmoe cohort are shown in Table 2. The GGT levels for quartiles 1, 2, 3, and 4 were ≤ 13, 14–17, 18–25 and ≥ 26 U/L, respectively. In univariate analysis stratified for cohort a statistically significant increase in risk for breast cancer was seen in woman with GGT-levels between 18–25 U/L (quartile 3) and ≥ 26 (quartile 4) compared to the reference GGT level of ≤13 U/L (quartile 1), respectively (HR 1.39, 95% CI 1.25 to 1.54 for quartile 3; HR 1.76, 95% CI 1.59 to 1.94 for quartile 4; p<0.001). In multivariate analysis (adjusted for the established breast cancer risk factors age, BMI and smoking status; stratified by cohort), women with GGT levels of ≥26 U/L (quartile 4) were at significantly higher risk for breast cancer compared to the quartile 1 GGT-group (HR 1.21, 95% CI 1.09 to 1.35; p = 0.005).

**Table 2:** Crude (univariate model) and adjusted risk (multivariate model I) estimates of breast cancer incidence in the pooled Vorarlberg and Malmoe cohort.

GGT levels	Breast cancer incidence, N (%)	Univariate model, HR (95% CI)	Multivariate model I, HR (95% CI)
Quartile 1* (≤13 U/L)	649 (2.2%)	Reference	Reference
Quartile 2* (14–17 U/L)	601 (2.5%)	1.10 (0.99 to 1.23)	1.02 (0.91 to 1.14)
Quartile 3* (18–25 U/L)	890 (3%)	1.39 (1.25 to 1.54)	1.11 (1 to 1.24)
Quartile 4* (≥26 U/L)	921 (3.8%)	1.76 (1.59 to 1.94)	1.21 (1.09 to 1.35)
p for trend (GGT)		< 0.001	0.005

CI confidence interval, HR hazard ratio, GGT γ-Glutamyltransferase, U/L units/Litre

Univariate model stratified by cohort. Multivariate model I adjusted for age, body mass index and smoking status stratified by cohort.

*Quartile size: Quartile 1 27.8%, Quartile 2 22.3%, Quartile 3 27.6%, Quartile 4 22.3%

In the Malmoe but not in the Vorarlberg cohort, data on alcohol consumption were available. Table 3 shows adjusted risk estimates of breast cancer incidence in the Malmoe cohort adjusted for age, BMI, smoking status, physical activity, parity and OC-use (multivariate model II) and additionally adjusted for alcohol (multivariate model III).

A statistically significant and almost identical increase in risk for breast cancer was seen in women with GGT-levels ≥26 U/L (quartile 4) compared to women with GGT levels ≤13 U/L (quartile 1) in both multivariate models showing almost identical hazard ratios and confidence intervals when excluding or including alcohol consumption (HR 1.39, 95% CI 1.13–1.71, p = 0.004 for model II; HR 1.37, 95% CI 1.11–1.69, p = 0.006 for model III).

In both analyses, pooled and Malmoe cohort only, there were no statistically significant interactions (all p-values for interactions >0.05) in the relationship of GGT with breast cancer incidence regarding age, BMI, smoking status, alcohol consumption and menopausal status (age groups i.e. < 50 or > 50 were used as proxy for menopausal status).

**Table 3:** Adjusted risk estimates of breast cancer incidence in the Malmoe cohort (multivariate model II) adjusted additionally for alcohol consumption (multivariate model III).

GGT levels	Breast cancer incidence, N (%)	Multivariate model II, HR (95% CI)	Multivariate model III, HR (95% CI)
Quartile 1* (≤13 U/L)	649 (2.2%)	Reference	Reference
Quartile 2* (14–17 U/L)	601 (2.5%)	1.11 (0.90 to 1.36)	1.01 (0.89 to 1.35)
Quartile 3* (18–25 U/L)	890 (3.0%)	1.09 (0.88 to 1.36)	1.08 (0.87 to 1.35)
Quartile 4* (≥26 U/L)	921 (3.8%)	1.39 (1.13 to 1.71)	1.37 (1.11 to 1.69)
**Alcohol consumption** **			
No	523 (6.7%)		Reference
Yes	205 (8.2%)		1.19 (1.01 to 1.41)
p for trend (GGT)		0.004	0.006

CI confidence interval, HR hazard ratio, GGT γ-Glutamyltransferase, U/L units/Litre. Model II adjusted for age, body mass index, smoking status, physical activity, parity, and oral contraceptive-use; Malmoe cohort only. Model III adjusted for age, body mass index, smoking status, physical activity, parity, oral contraceptive-use and alcohol consumption; Malmoe cohort only

*Quartile size: Quartile 1 29.1%, Quartile 2 25.3%, Quartile 3 22.3%, Quartile 4 23.3%

** Questionnaire issue: “Do you mainly drink alcohol at weekends or public holidays?”

### Cross-sectional correlates of GGT with breast cancer risk factors

Women in the Malmoe Cohort have filled in a detailed questionnaire on anamnestic data covering a high number of established breast cancer risk factors and other parameters at the time point of cohort inclusion. Significant correlations found between anamnestic parameters and GGT levels in the Malmoe cohort are shown in Table 4. High GGT levels were significantly associated with high age (p<0.001), high BMI (p<0.001), current or former smoking status (p<0.001), oral contraception use (p<0.001), alcohol consumption (p<0.001) and treatment for nervous and mental disorders (p = 0.02). A statistically significant, inverse correlation was seen between GGT levels and physical activity (p = 0.001) and parity (p = 0.008), respectively.

**Table 4:** Multiple linear regression of GGT-levels in logarithmic units with established risk factors for breast cancer and other anamnestic parameters in the Malmoe cohort for complete cases (n = 6441).

Risk factor / Descriptive statistics	N (%), Mean (± SD)	Standardized Beta	p-value
Age in completed years	51.7 (±4.5)	0.222	<0.001
Body mass index (kg/m^2^)	24.8 (±4.3)	0.086	<0.001
Smoking status*		0.091	<0.001
No	4,430 (66.7%)		
Yes	2,211 (33.3%)		
Physical activity***		-0.038	0.001
No	1,700 (25.6%)		
Yes	4,941 (74.4%)		
**Parity**		-0.032	0.008
Nulliparous	1,076 (16.2%)		
Other	5,565 (83.8%)		
**Oral contraceptive use**		0.075	<0.001
No	6,326 (95.3%)		
Yes	315 (4.7%)		
Alcohol consumption**		0.067	<0.001
No	5,032 (75.8%)		
Yes	1,609 (24.2%)		
**Treatment for nervous or mental disorders**		0.028	0.02
No	5,708 (86%)		
Yes	933 (14%)		

Categorical variables were included as 0/1 coded indicator variables into the regression model.

* Questionnaire issue: “Do you smoke or have you been smoking?”

** Questionnaire issue: “Do you mainly drink alcohol at weekends or public holidays?”

*** Questionnaire issue: “Are you aerobic physical active at least for 150 minutes (moderate-intensive) or at least for 75 minutes (vigorous-intensive) or an equivalent combination of moderate- to vigorous-intensive active per week?”

## Discussion

The present study investigates the relationship between GGT and breast cancer risk in a large-scale population-based pooled cohort study considering established breast cancer risk factors including life style related parameters. Our data suggest a statistically significant association between GGT and the development of breast cancer, persisting after adjustment for several confounding factors under different modeling strategies during a median follow-up period of 17.5 years. Importantly, GGT remained an independent risk factor for breast cancer when adjusting for alcohol intake. In clinical routine, GGT values below 36 U/L are considered normal, discriminating between normal low (<17.99 U/L) and normal high values (18–35.99 U/L) [20, 21]. Notably, our multivariate models identified GGT values in the range of ≥ 26 U/L significantly increased breast cancer risk.

Only two previous studies have specifically focused on the relationship of serum GGT-levels with the site-specific risk of breast cancer [13, 14].

Fentiman et al. prospectively followed a relatively small cohort of 1,083 healthy women in Guernsey UK [13]. During the follow-up 96 women developed breast cancer. They found a highly significant relationship between GGT and breast cancer risk with hazard ratios of >2 for quartiles three and four in comparison to quartile one in a multivariate model. The present large population-based investigation in more than 107,000 women and 3,179 incident breast cancer cases confirms their finding that serum-GGT level is a significant risk factor for breast cancer. Fentiman et al. observed this association only in premenopausal women, whereas we did not see a significant interaction between GGT and breast cancer incidence with respect to menopausal status when using age groups as a proxy. The multivariate breast cancer model in the Fentiman study considered the breast cancer risk factors age, age at menarche, age at first birth/nulliparity, height and weight. However, classic lifestyle risk factors such as, smoking, physical activity or alcohol consumption were unknown [13].

Van Hemelrijck et al. studied the association of GGT with increased risk of overall cancer incidence and several site-specific malignancies in women and men in a large Swedish cohort (AMORIS study) [14]. They showed a significant association of GGT levels with increased incidence of pooled female malignancies (combined breast and female genital malignancies). In a site-specific subanalysis in 5626 incident breast cancer cases adjusted for age, socio-economic status and history of circulatory disease, a significant association between GGT and breast cancer risk was seen. The risk was more pronounced in women with high blood glucose compared to normal blood glucose levels. One can speculate that high glucose levels in these women were indicative for obesity. The combined effect of two risk factors may explain the higher individual risk for breast cancer development. However, BMI and other classic breast cancer risk factors were unknown in this study [14].

In contrast to previous studies, the present study independently evaluates GGT and breast cancer risk considering in particular life-style factors. We addressed the question, whether elevated serum GGT levels reflect independently an increased risk for breast cancer beyond the risk factor alcohol. Our data clearly suggest a role of GGT in breast cancer carcinogenesis beyond alcohol intake. Fentiman et al. state that the blood samples in their study were collected between 1986 and 1990 before there was a major increase in alcohol use in women [13]. Van Hemelrijck et al. indirectly accounted for liver dysfunction by stratifying the association between GGT and cancer risk by alanine aminotransferase (ALT) levels. The liver enzyme ALT is a specific biomarker for liver damage. Stratified analysis did not show any difference by ALT levels, suggesting that GGT has an independent role in cancer risk form liver dysfunction [14].

The study of Tsuboya et al. specifically investigated the effect of alcohol consumption on the relationship between GGT levels and overall-/site-specific cancer incidence in a Japanese cohort of 15,032 participants (thereof 8,659 women) [22]. Among participants in the highest quartile, a significant association between GGT and overall cancer incidence independent of alcohol consumption was found in a multivariate model. In cancer-site specific subanalyses, GGT remained an alcohol-independent risk factor for colorectal and liver cancer. In other alcohol-related cancers such as breast, pancreatic and oesophageal cancer, but not in non-alcohol-related cancer sites, GGT hazard ratios where increased but did not reach statistical significance. Tsuboya et al. concluded that the positive associations seen in alcohol-related cancers might possibly be due to residual confounding by alcohol [22]. They found their hypothesis supported by the finding that a positive trend for an association between GGT and overall cancer incidence was observed in current drinkers, but not in ever drinkers. However, previous findings in the Vorarlberg cohort and the AMORIS study strongly speak against this theory, showing a significant impact of GGT on cancer incidences unrelated to alcohol consumption such as malignancies of the respiratory system/intrathoracic organs, urinary organs, male genital organs and hematologic malignancies in women [11, 12, 14]. An independent role of GGT in carcinogenesis is also in line with experimental models suggesting a more general causative of GGT with carcinogenesis [3, 4]. Finally, when focusing on GGT and breast cancer, it is noteworthy that data in the study of Tsuboya et al. are based on only 71 incident breast cancer cases among 8,659 women [22]. This possibly also reflects the well-known lower breast cancer rate of Japanese women compared to women in Western countries. Together with the fact that alcohol tolerability also differs in the Asian and European population [23], the comparability of data might be jeopardized and separate studies are warranted.

The exact underlying mechanism linking GGT to carcinogenesis is unclear. Traditionally, GGT and GSH have been regarded as essential components of the cell’s defence apparatus against oxidative stress [1]. However, the dysregulation of GGT in several tumours raised the question whether increased GGT expression itself has an active role in neoplastic transformation [24]. Experimental evidence has elucidated the ability of GGT to modulate crucial redox-sensitive functions, such as antioxidant defenses and cellular proliferative and apoptotic balance [24, 25]. The presence of elevated GGT levels seems to reflect a state of persistent oxidative stress as part of the biological pathway related to cancer development [26, 27]. Furthermore, in clinical studies a pivotal role of GGT in tumour invasion, progression and drug resistance has repeatedly been suggested [2–4, 28].

A major strength of the present study lies in the consideration of established breast cancer risk factors and in the prospective evaluation of GGT in a large number of women with particular long time follow-up. However, detailed information on some risk factors, including e.g. alcohol consumption and physical activity, were available only in the Malmoe cohort, which is a clear limitation of our study. Furthermore some other confounding factors, e.g. hormone replacement therapies, were completely missing.

## Conclusions

In summary, this is the first study evaluating GGT for site-specific risk of breast cancer in a large cohort of women considering established breast cancer risk factors including alcohol intake. Our data clearly confirm GGT as a significant risk factor for breast cancer and suggest a role in breast cancer carcinogenesis independent of alcohol consumption.
